# *Salvia miltiorrhiza* bunge extracts: a promising source for anti-atopic dermatitis activity

**DOI:** 10.1186/s12906-024-04524-z

**Published:** 2024-06-06

**Authors:** Da Hye Ryu, Jwa Yeong Cho, Hyung-Seok Yu, Jin-Woo Kim, Jin-Chul Kim, Yang-Ju Son, Chu Won Nho, Muhammad Hamayun, Ho-Youn Kim

**Affiliations:** 1 Smart Farm Research Center, Korea Institute of Science and Technology (KIST), Gangneung, Gangwon 25451 Republic of Korea; 2 Division of Bio-Medical Science and Technology, KIST School, Korea University of Science and Technology (UST), Daejeon, 34113 Republic of Korea; 3 Natural Product Informatics Center, Korea Institute of Science and Technology (KIST), Gangneung, 25451 Republic of Korea; 4 Interdisciplinary Program in Agricultural Genomics, Seoul National University, Seoul, 08826 Republic of Korea; 5 Department of Food and Nutrition, College of Biotechnology and Natural Resources, Chung-Ang University, Anseong, 17546 Republic of Korea; 6 Department of Botany, Abdul Wali Khan University Mardan, Garden Campus, Khyber Pakhtunkhwa, 23200 Pakistan

**Keywords:** *Salvia miltiorrhiza* Bunge, Antioxidant, Anti-inflammatory, Anti-atopic dermatitis, UPLC-TQ-MS/MS, HPLC-DAD, Phenolics, Tanshinones, Bio-guided fractionation

## Abstract

**Background:**

Atopic dermatitis (AD) is a chronic inflammatory condition characterized by the accumulation of reactive oxygen species and the expression of inflammatory factors. Regarding its anti-atopic activity, numerous traditional medicinal materials and secondary metabolic products play pivotal roles in modulating the associated mechanisms.

**Methods:**

This study aimed to utilize *Salvia miltiorrhiza* Bunge (SMB) as an anti-AD source. In-vitro activity assessments and qualitative and quantitative analyses using UPLC-TQ-MS/MS and HPLC-DAD were conducted in two cultivars (‘Dasan’ and ‘Kosan’). Statistical analysis indicated that the profiles of their secondary metabolites contribute significantly to their pharmacological properties. Consequently, bio-guided fractionation was undertaken to figure out the distinct roles of the secondary metabolites present in SMB.

**Results:**

Comparative study of two cultivars indicated that ‘Dasan’, having higher salvianolic acid A and B, exhibited stronger antioxidant and anti-inflammatory activities. Meanwhile, ‘Kosan’, containing higher tanshinones, showed higher alleviating activities on anti-AD related genes in mRNA levels. Additionally, performed bio-guided fractionation re-confirmed that the hydrophilic compounds of SMB can prevent AD by inhibiting accumulation of ROS and suppressing inflammatory factors and the lipophilic components can directly inhibit AD.

**Conclusions:**

SMB was revealed as a good source for anti-AD activity. Several bioactive compounds were identified from the UPLC-TQ-MS/MS and different compounds content was linked to biological activities. Characterization of these compounds may be helpful to understand differential role of secondary metabolites from SMB on alleviation of AD.

**Supplementary Information:**

The online version contains supplementary material available at 10.1186/s12906-024-04524-z.

## Background

Atopic dermatitis (AD) is a multifactorial inflammatory skin disease characterized by pruritus, facial and extensor eczema in childhood, and chronicity of dermatitis [1]. The lifetime prevalence of AD is 10–20% in early infancy and childhood, primarily due to relatively low immunity, with 45% of cases occurring during the first 6 months. In contrast, the prevalence is 1–3% in adults [2]. AD has a lower prevalence in predominantly rural or agricultural areas with limited exposure to harmful environments compared to industrialized countries [1]. However, with the development of industry, the incidence of AD has increased due to environmental triggers, such as increased exposure to sensitizing allergens and decreased immunity caused by parasitic and microbial components [3]. Over the past three decades, there has been a 2 to 3-fold increase in the incidence rate of AD in industrial areas [1], highlighting it as a substantial global public health concern [4].

Over the past three decades, there has been a two to threefold increase in the incidence rate of AD in industrial areas, highlighting it as a significant global public health concern [4]. Concerning the safety issues of developed synthetic alternatives, there has been a growing demand for natural therapies employing medicinal materials with antioxidant properties [5, 6]. Oxidative stress, triggered by reactive oxygen species (ROS), can compromise skin integrity. This increased skin permeability, resulting from damage to the compromised skin barrier, facilitates the entry of allergens and pathogens, leading to conditions like facial and extensor eczema [7, 8]. Therefore, potent antioxidants can counteract ROS overproduction and potentially enhance skin barrier formation by improving epidermal differentiation [9].

In this study, *Salvia miltiorrhiza* Bunge (SMB) was selected as a potential plant for the treatment of AD due to its strong antioxidant properties [10] and its traditional medicinal use in treating skin diseases [11]. SMB belongs to the genus Salvia of the Lamiaceae family [12] and is a well-known Traditional Chinese Medicine used for various purposes, including the treatment of hemorrhages, menstrual irregularities, and the promotion of blood flow [13]. It has been reported to exhibit remarkable activities in scavenging reactive oxygen species [14, 15], anti-diabetic effects [16], inhibition of inflammation, and suppression of cancer growth [17–19]. These pharmacological properties are believed to be linked to the secondary metabolites of SMB. To date, 40 abietane diterpenes, known as tanshinones, and 50 phenolic acids have been identified in SMB [20], which are known to contribute to various immune-related functions [21, 22]. The diterpene components found in SMB exhibit lipophilic properties. Among these components, tanshinone IIA is recognized as the primary constituent and serves as a major component and quality indicator. Moreover, tanshinone IIA has been linked to notable antioxidant effects, potentially impeding the interaction between lipid peroxidation products and DNA within hepatic cells [23]. Another primary component within the group of functional compounds is salvianolic acid B, which also exhibits potent natural antioxidant properties. Its antioxidant ability has been demonstrated to reduce malondialdehyde levels and alleviate brain and heart damage in animal experiments [24, 25].

In Korea, efforts have been made to address challenges such as high import dependence on China and the low-quality uniformity of *Salvia miltiorrhiza* Bunge. Two cultivars of SMB with high levels of functional compounds have been successfully developed. In 2015, ‘Dasan’ (DS), the first Korean SMB cultivar with improved functionality and productivity from native species, was developed, and ‘Kosan’ (KS) was further developed in 2018. To enhance the potential of SMB as a natural source for treating AD, this study utilized both Korean SMB cultivars to evaluate their inhibitory activity on various inflammatory factors associated with AD. Additionally, the biological activities related to AD, such as antioxidant and anti-inflammatory activities, were confirmed.

## Methods

### Reagents

For the in-vitro assays, 2,2-diphenyl-1-picrylhydrazyl (DPPH) and 2,2’-azino-bis-3-ethylbenzothiazoline-6-sulfonic acid (ABTS) were purchased from Sigma-Aldrich (St. Louis, MO, USA). For the in-vivo assays, Dulbecco’s modified Eagle’s medium (DMEM), fetal bovine serum (FBS), phosphate-buffered saline (PBS), an antibiotic solution (containing 10,000 U/ml of penicillin and 10,000 µg/ml of streptomycin), a 0.25% trypsin-EDTA solution, and Dulbecco’s phosphate-buffered saline (DPBS) were purchased from Hyclone Laboratories, Inc. (South Logan, UT, USA). Recombinant human tumor necrosis factor (TNF)-α and interferon (IFN)-γ were acquired from PeproTech (Rocky Hill, NJ, USA). Other chemicals, including 3-(4,5-dimethylthiazol-2-yl)-2,5-diphenyltetrazolium bromide (MTT) and dimethyl sulfoxide (DMSO), were obtained from Sigma-Aldrich (St. Louis, MO, USA).

### Sample preparation

Two cultivars of SMB (DS and KS), cultivated in Soi-myeon, Eumseong-gun, Chungcheongbuk-do, Republic of Korea, were provided by the Rural Development Administration of Korea. SMB was harvested on March 25, 2021, and frozen using liquid nitrogen. The samples were then dried using a freeze dryer for 5 days and finely ground. For high-performance liquid chromatogram (HPLC) analysis and the assessment of antioxidant and anti-inflammatory activities, the dried samples were extracted with methanol using a reflux extractor for 2 h at 50 °C. The extracts were filtered through a 0.22 μm membrane filter (PVDF syringe filter, hydrophobic, 13 mm diameter, 0.22 μm pore size, Whatman International, Maidstone, UK), concentrated using a rotary evaporator equipped with a circulating water vacuum pump, and re-dissolved in DMSO to a concentration of 40 mg/mL. They were then stored at -80 °C before the experiment.

### Total phenolics content (TPC)

The total phenolic content (TPC) was measured using a modified method [26]. In a 96-well plate, 10 µl of the sample was mixed with 200 µl of 2% Na_2_CO_3_ for 3 min. The mixture was then reacted with 10 µl of 1 N Folin-Ciocalteu’s reagent for 27 min, and the resulting absorbance was measured at 750 nm using a multi-detection microplate reader (Synergy HT; BioTek Instruments, Winooski, VT, USA). TPC was calculated using a calibration curve (Y = 2.4297X + 0.0008, *R*^*2*^ = 0.9986) generated with various concentrations of gallic acid (ranging from 31.25 to 1000 µg/ml). The TPC was expressed as gallic acid equivalents (GAE) in milligrams per gram (mg/g).

### Effect of extracts on antioxidant activities

The DPPH radical scavenging activity was assessed using the DPPH decolorization method with some modifications, as per MJ Park, DH Ryu, JY Cho, DG Lee, JN Lee and Y-H Kang [27]. Before the experiment, DPPH powder was dissolved in ethanol to a concentration of 0.2 mM, and its absorbance was adjusted to 1.00 ± 0.05. Then, 20 µl of samples with varying concentrations were added and mixed with the prepared DPPH solution (180 µl) in 96-well plates for 30 min. The absorbance was measured at 517 nm using the multi-detection microplate reader, Synergy HT (BioTek Instruments, Winooski, VT, USA), and the reduction values were expressed as the RC_50_ value, which represents the concentration required to reduce 50% of the radicals.

The ABTS free radical scavenging activity was determined using the decolorization method with some modifications, following the procedure described by DH Ryu, JY Cho, NB Sadiq, JC Kim, B Lee, M Hamayun, TS Lee, HS Kim, SH Park, CW Nho, et al. [28]. ABTS stock solution was diluted with 2.45 mM K_2_S_2_O_8_ and stored at 4 °C overnight to produce the radical cation. The stabilized ABTS solution was mixed with ethanol to adjust the absorbance to 0.80 ± 0.05. After preparation, 20 µl of the sample was reacted with 180 µl of the ABTS solution for 5 min at room temperature, and the change in absorbance was measured at 734 nm using a multi-detection microplate reader. The activity was expressed as the RC_50_ value, representing the concentration required to eliminate 50% of the radicals.

### Effect of extracts on nitric oxide production in raw 264.7 cell

The Raw 264.7 cell line was obtained from the American Type Culture Collection (ATCC; Rockville, MD, USA). Raw 264.7 cells were cultured in a humidified incubator with 5% CO_2_ at 37 °C, using DMEM media containing 10% FBS and 1% antibiotic solution.

For investigation of cell viability, The Raw 264.7 cells were seeded in a 96-well plate at a density of 5 × 10^4^ cells per well and incubated for 24 h. After incubation, the supernatant media was removed, and the cells were washed with PBS. Subsequently, 100 µl of fresh culture media containing varying concentrations of DS or KS extracts (ranging from 6.25 to 100 µg/ml) were added to the wells. Cell viability was determined using an MTT assay 24 h later. In brief, 10 µl of MTT solution was added to each well and left for 4 hours. After this incubation, the media was aspirated, and 100 µl of DMSO was added to the wells. After 30 min, the absorbance was measured at 570 nm.

At non-toxicity concentrations, the effects of extracts on inhibiting nitric oxide (NO) production in Raw 264.7 cells were measured. The Raw 264.7 cells were seeded at a density of 5 × 10^4^ cells per well in a 96-well plate. After 24 h of incubation, the supernatant media was aspirated, and the plate was washed with PBS. Subsequently, 50 µl of culture media containing SMB samples (at concentrations of 20–50 µg/mL) or without SMB samples was added to the wells and incubated for 30 min. Afterward, 50 µl of media with or without lipopolysaccharide (LPS) at a concentration of 2 µg/ml was added to the wells. The cells were then incubated for 24 h, and the supernatant media was collected for the detection of NO content. A total of 40 µl of the collected media was mixed with an equal volume of Griess reagent and incubated for 20 min. The absorbance of the mixture was measured at 550 nm, and the NO content in the media was determined using a standard calibration curve of sodium nitrite (Y = 0.0029X + 0.0406; *R*^*2*^ = 0.9952).

### Effects of extracts on the expression of cytokines and chemokines in the HaCaT cell

The human keratinocyte HaCaT cell line (obtained from Cell Lines Service GmbH, Eppelheim, Germany) was cultured in a growth medium composed of DMEM supplemented with 10% FBS and 1% antibiotic solution. The cells were maintained at 37 °C in a humidified atmosphere containing 5% CO_2_. Sub-culturing was performed when the cells reached 80–90% confluence, and this process was carried out within the first 10 passages.

Cell viability was determined using the MTT assay as previously described by H-S Yu, W-J Kim, W-Y Bae, N-K Lee and H-D Paik [29], with minor modification. HaCaT cells were seeded in 96-well culture plates at a density of 1 × 10^4^ cells per well and incubated for 24 h. Subsequently, the cells were treated with various concentrations of samples. After an additional 24 h of incubation, the supernatant in each well was replaced with a growth medium containing 0.5 mg/ml of MTT, and the cells were incubated for an additional 2 h. Following this, the supernatant was removed, and the formazan deposits generated in each well were dissolved by adding 200 µl of DMSO. The absorbance was measured at 570 nm. Cell viability was calculated as a percentage by comparing the absorbance with that of the control groups, with cells not treated with samples defined as the control group.

The effect of the samples on inflammation-associated mRNA expression was assessed through qRT-PCR analysis, as described by H-S Yu, N-K Lee, A-J Choi, J-S Choe, CH Bae and H-D Paik [30]. Cells were plated in 6-well culture plates at a density of 1.5 × 10^5^ cells per well and incubated for 48 h. They were then subjected to serum starvation by using DMEM supplemented with 0.5% FBS and 1% antibiotic solution for 24 h. Subsequently, the supernatant was replaced with fresh growth media containing different concentrations of samples (0 and 10 µg/ml) and incubated for an additional 1 h. The cells were then stimulated with or without TNF-α/IFN-γ (10 ng/mL each) for 24 h. After being rinsed thrice with ice-cold DPBS, total RNA was extracted using the RNeasy Mini Kit (Qiagen, Hilden, Germany). An aliquot of total RNA (1 µg) was reverse-transcribed into cDNA using the SensiFASTTM cDNA synthesis kit (Bioline, London, UK). The SensiFASTTM SYBR Hi-ROX PCR kit (Bioline) was used for qRT-PCR analysis. The PCR mixture, consisting of SYBR Green PCR Master Mix (10 µl), 400 µM of specific primers (0.8 µl), synthesized cDNA (2 µl), and water (6.4 µl), was amplified as follows: denaturation at 95 °C for 2 min, followed by 40 cycles at 95 °C for 5 seconds, 60 °C for 20 s, and 72 °C for 20 s. Amplification of a single product was confirmed with the melting curve. The relative mRNA expression levels of each target gene were analyzed using the 2^−(ave.ΔΔCT)^ method after normalization with GAPDH. The sequences of each primer used in this qRT-PCR analysis are provided in Table 1.

**Table 1:** Primer sequences used in quantitative real-time polymerase chain reaction

Gene	Primer sequence (5’ → 3’)
GAPDH	Forward: GTGATGGCATGGACTGTGGT
	Reverse: AAGGGTCATCATCTCTGCCC
TARC	Forward: ACTGCTCCAGGGATGCCATCGTTTTT
	Reverse: ACAAGGGGATGGGATCTCCCTCACTG
MDC	Forward: AGGACAGAGCATGGCTCGCCTACAGA
	Reverse: TAATGGCAGGGAGCTAGGGCTCCTGA
MCP-1	Forward: AGTCTCTGCCGCCCTTCTGTG
	Reverse: TGCTGCTGGTGATTCTTCTAT
IL-6	Forward: ACCTGAACCTTCCAAAGA
	Reverse: TTCCTCACTACTCTCAAATCT
IL-8	Forward: AGGGTTGTGGAGAAGTT
	Reverse: GGCATCTTCACTGATTCTTG

### Qualitative analysis and quantitative of major compounds

To qualitatively analyze the secondary metabolites of SMB, UPLC-TQ-MS/MS was performed. The SMB samples were analyzed using an Agilent 1290 Infinity II LC system (Agilent, Waldbronn, Germany) coupled with an Agilent 6470 B Triple Quadrupole instrument. The injection volume was set at 1 µL, and the injected sample was carried out at a column temperature of 45 °C using a YMC-Triart C18 column (100 × 2.0 mm, I.D. S-1.9 μm, 8 nm). The mobile phase consisted of water containing 0.2% formic acid (A) and acetonitrile containing 0.2% formic acid (B). The flow rate of the mobile phase was set at 350 µL per minute, and a gradient system was applied for the analysis. Initially, the B ratio was set at 10% and maintained for 1 min. Afterward, the B ratio was increased to 40% at 8 min and 50% at 20 min. Finally, the B ratio was increased to 100% at 40 min, and there was a 2-minute post-run for column washing. The detection wavelength was set at 280 nm, and the detected peaks were further characterized using UPLC-TQ-MS/MS analysis. MS and MS/MS detection was carried out using an Agilent 6470B Triple Quadrupole instrument. The operating conditions were set as follows: drying gas (N2) flow rate at 7 L per minute, drying gas temperature at 325 °C, nebulizer at 25 psi, sheath gas flow rate at 7 L per minute, sheath gas temperature at 250 °C, capillary at 3500 V, fragmentor at 135 V, collision energy at 25 V, and scan mode in both MS scan and product ion mode. The mass spectra were recorded within a range of m/z 100–1000 in negative mode and m/z 100–500 in positive mode. Data acquisition and processing were performed using the Agilent Mass Hunter Workstation Acquisition Software Version B.05.01 and Qualitative Analysis Software Version B.07.00.

The main compounds of SMB were quantified using HPLC equipment (Agilent 1200, Agilent, Waldbronn, Germany). A 10 µL sample was injected and loaded onto a YMC C18 Triart column maintained at 40 °C. The compounds were separated using a gradient system with two mobile phases (A: water containing 0.2% formic acid and B: acetonitrile containing 0.2% formic acid) as follows: 0 min, 20% B; 5 min, 20% B; 17 min, 50% B; 23 min, 80% B; 30 min, 90% B; 40 min, 100% B; 43 min, 100% B; 47 min, 20% B; and 50 min, 20% B. The peaks were detected at 280 nm and quantified based on the calibration curve.

### Activity-guided fractionation

Heat-dried SMB (600 g) was subjected to ultrasound-assisted extraction (UAE) using methanol at 60 °C for 1 h, with a total of five extraction cycles. The resulting extracts were concentrated under vacuum using a rotary evaporator, yielding the methanol extract (ME; 120 g), which was then suspended in 2 L of distilled water. This dissolved extract was subsequently transferred into a separatory funnel and sequentially fractionated using 3 L of hexane (10 times), 2 L of ethyl acetate (10 times), and 2 L of butanol (10 times). This fractionation process resulted in the isolation of five fractions: the methanol extract (ME), hexane fraction (HF), ethyl acetate fraction (EF), butanol fraction (BF), and water fraction (WF), as illustrated in Fig. S1.

The chromatogram, as shown in Fig. S2, obtained through HPLC analysis, exhibited a distinct profile for each fraction, indicating successful fractionation. The presence of different major components in each fraction was confirmed.

### Verification of the activity of the fractionation layers

The obtained fractions were subjected to activity validation, including antioxidant activity assessed through DPPH and ABTS assays (as described in Sect. 2.3 and 2.4). The anti-AD activity was confirmed through the analysis of AD-related gene expression using RT-qPCR (as detailed in Sect. 2.10). Additionally, their secondary metabolites were quantified using the HPLC-DAD method (as outlined in Sect. 2.11).

### Statistical analysis

The SMB extracts were analyzed using UPLC-TQ-MS/MS and subsequently subjected to various biological activity assays. Experimental data are presented as means ± standard deviation. For statistical analysis, analysis of variance (ANOVA) was conducted using Pearson’s method to assess the differences between samples. The statistical significance of the results was denoted as follows: * for *p* < 0.05, ** for *p* < 0.01, *** for *p* < 0.005, and # for *p* < 0.001. Principal component analysis (PCA) was employed to analyze all datasets comprehensively without data loss and to enhance interpretability, utilizing the SIMCA software (version 15.0.2, Sartorius).

## Results

### Antioxidant activity

**Fig. 1:**
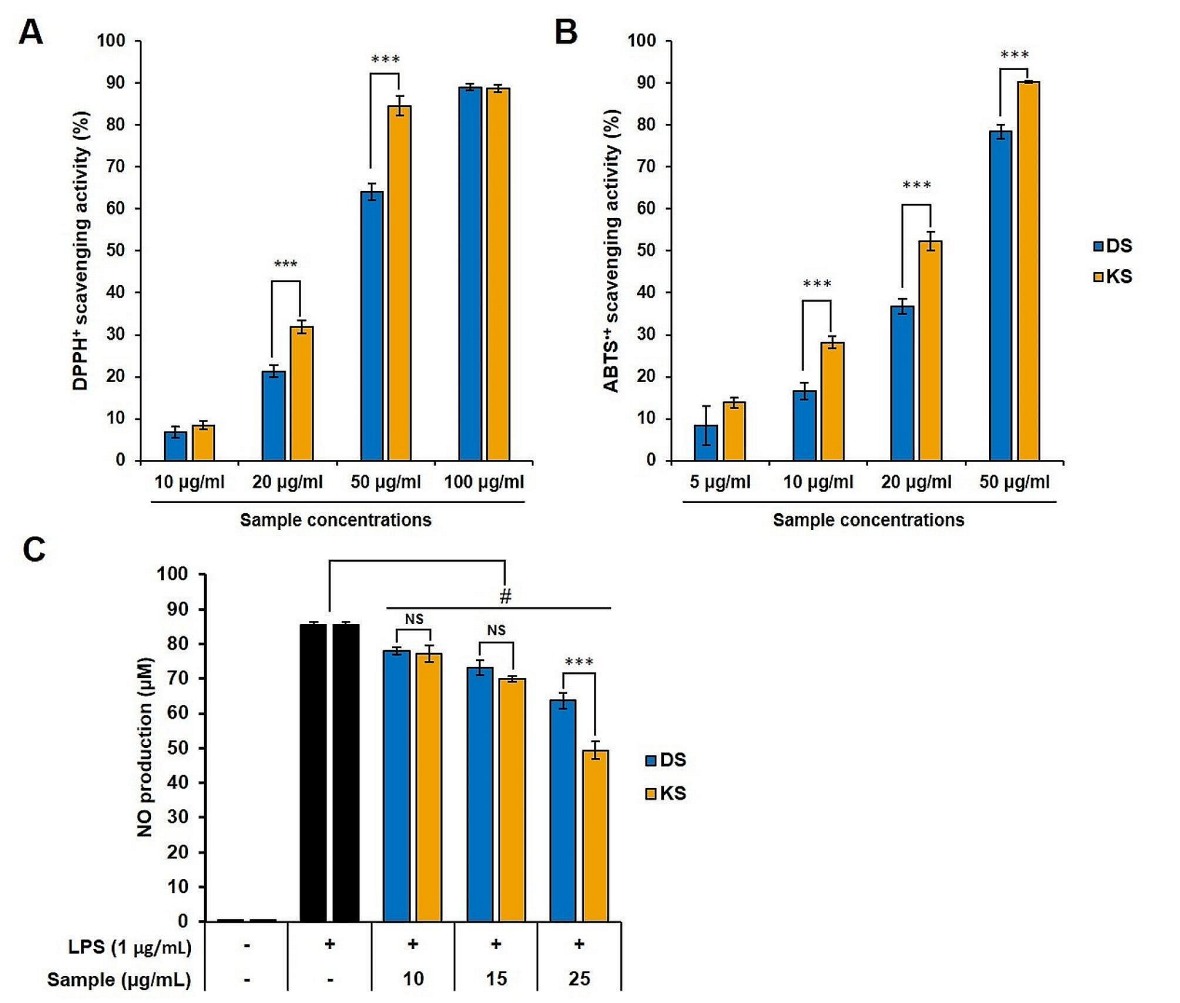
Antioxidant activities measured by DPPH assay (**A**) and ABTS assay (**B**) in diverse concentrations. Inhibition activity for nitric oxide (NO) production (**C**) of DS and KS extracts were evaluated for confirming anti-inflammatory activity. Data was expressed by 3 determinations ± standard deviation and significant difference in same concentration was determined using Student’s t-test and expressed following the *p* value as follows: *, *p* < 0.05; **, *p* < 0.01; ***, *p* < 0.005; and #, *p* < 0.001. # indicate significant difference between positive control (the NO production in the LPS stimulated RAW 264.7 cell) and samples at *p* < 0.005

The antioxidant activity of SMB extracts was evaluated and is depicted in Fig. 1. Significant differences (*p* < 0.05) in activities between cultivars were observed. The DPPH assay results indicated that there were no significant differences in antioxidant activity at the lowest concentration (10 µg/ml) and the highest concentration (100 µg/ml). However, when comparing sample concentrations ranging from 20 µg/ml to 50 µg/ml, higher DPPH radical scavenging activity was observed in KS (31.8 ± 1.5% and 84.5 ± 2.3%) compared to DS (21.3 ± 1.4% and 64.1 ± 2.1%) (Fig. 1A). Similarly, the ABTS assay results showed a similar trend to the DPPH assay, but the activity values were higher when comparing RC_50_ values: DS DPPHRC_50_ = 50.2 ± 0.7 µg/ml; DS ABTSRC_50_ = 30.9 ± 0.8 µg/ml; KS DPPHRC_50_ = 31.2 ± 0.8 µg/ml; and KS ABTSRC50 = 23.6 ± 0.4 µg/ml, respectively. The ABTS radical scavenging activity exhibited a concentration-dependent increase, and at the same concentration, relatively higher ABTS radical scavenging activity was observed in KS compared to DS (Fig. 1B). Notably, at the lower concentrations, a greater difference in activity between cultivars was observed, with 1.7-, 1.4-, and 1.2-fold differences at 10, 20, and 50 µg/ml, respectively, which were statistically significant (*p* < 0.05) according to the T-test.

### Inhibition activity against NO production in raw 264.7 cells

The anti-inflammatory activity was assessed by measuring the inhibition of NO production in Raw 264.7 cells at non-toxic concentrations (10–25 µg/ml). The level of NO in the LPS-induced cells showed a significant increase (approximately 165.8-fold) compared to that in non-treated cells (*p* < 0.001), and the standard curve generated using nitrate as the reference standard is shown in Fig. 1C. The results in Fig. 1C demonstrate that SMB led to a reduction in NO levels in a dose-dependent manner, and KS exhibited stronger anti-inflammatory activity compared to DS. This observation supports the notion that anti-inflammatory activity is related to antioxidant activity, consistent with previous studies [31, 32].

### Evaluation of the inhibitory ability of AD-related factors by RT-qPCR

**Fig. 2:**
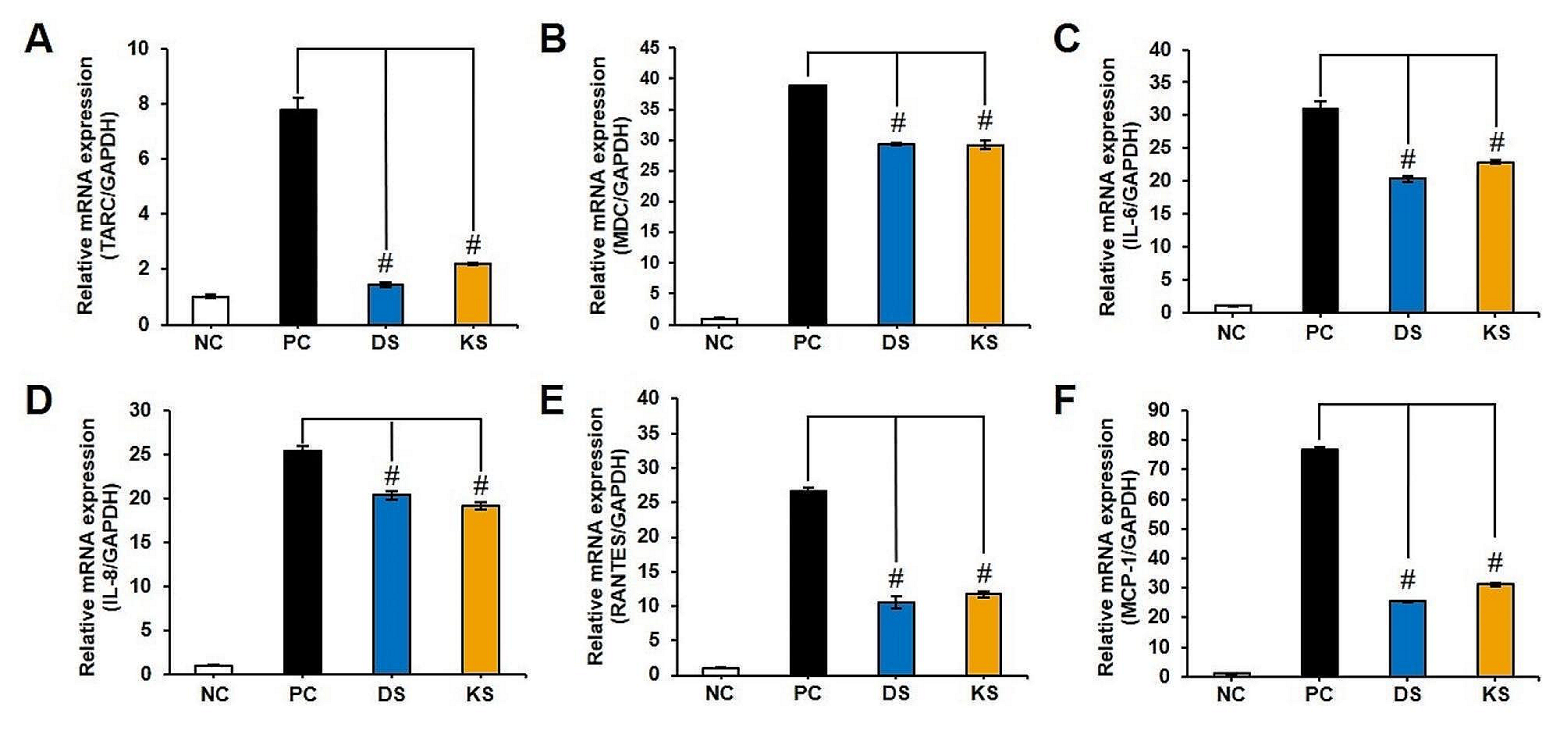
Effect of crude extract of SMB including DS and KS on TNF-α and IFN-γ-induced chemokines including TARC (**A**), MDC (**B**), IL-6 (**C**), IL-8 (**D**), RANTES (**E**), and MDC (**F**) in skin cell line (HaCaT cells) at the mRNA expression. #: *p* < 0.001

In addition to evaluating the anti-inflammatory activity in the normal macrophage cell line (Raw 264.7 cell), the anti-atopic dermatitis activity was assessed in the human epithelial keratinocyte cell line (HaCaT cell) using RT-qPCR. Before the experiment, cell viability was determined by the MTT assay, and non-toxic concentrations (10 µg/ml) were applied for the anti-atopic dermatitis activity assay. In this assay, HaCaT cells were stimulated by TNF-α and IFN-γ, and the mRNA expression of various inflammatory factors was measured and calculated relative to the GAPDH gene. As depicted in Fig. 2, these factors exhibited a significant reduction compared to the positive control (*p* < 0.001) at the mRNA level. This suggests that SMB can be a valuable source for the prevention and treatment of atopic dermatitis, with DS showing higher activity in inhibiting factors such as TARC (thymus and activation-regulated chemokine; CCL17), RANTES (regulated on activation, normal T-cell expressed and secreted; CCL5), MDC (macrophage-derived chemokine; CCL22), MCP-1 (monocyte chemoattractant protein-1), IL-6 (interleukin-6), and IL-8 (interleukin-8). Confirmation of whether it also affects actual protein expression was conducted through western blotting (followed Supplementary Method) and data was presented in Fig. S3. Remarkably, expression inhibition in the STAT pathway, known to be highly associated with the onset of atopic conditions, was clearly observed with concentration dependent manner. Given that the STAT (signal transducer and activation of transcription) pathway is the core signaling cascade for diverse inflammatory cytokines and growth factors, signal transduction, and subsequent regulation of gene transcription [33, 34], the activity of SMB against AD has been demonstrated.

### UPLC-TQ-MS/MS analysis

Through UPLC-TQ-MS/MS analysis, 12 compounds were tentatively identified based on their reported spectral data and published papers. The fragmentation pattern is illustrated in Fig. S4, and the peaks are listed in Table 2. Among these 12 compounds, six were polar compounds (phenolics) identified in negative ion mode, while the others were non-polar compounds (terpenoids) identified in positive ion mode. Peak 1 (Rt: 3.405 min) was identified as danshensu (m/z 198, C_9_H_10_O_5_) and exhibited m/z 135 corresponding to the loss of the carboxyl group (-COOH) and hydroxyl group (-OH), and m/z 123 due to carbon loss (12 Da). Peak 2 (Rt: 4.909 min) showed product ions at m/z 119 by the loss of hydroxyl (-OH) and was identified as protocatechualdehyde (m/z 138, C_7_H_6_O_3_). Lithospermic acid (Rt: 9.364 min, m/z 538, C_27_H_22_O_12_) exhibited fragment ions at m/z 339 and 197, corresponding to cleavage at C-9 and McLafferty rearrangement. Peak 4 (Rt: 10.644 min) was identified as rosmarinic acid (m/z 360, C_18_H_16_O_8_) by exhibiting caffeic acid (m/z 180, C_9_H_8_O_4_) and 3,4-dihydroxy phenyl lactic acid (m/z 198, C_9_H_10_O_5_) in protonated form. Meanwhile, salvianolic acid B, a form of rosmarinic acid dimer, exhibited product ions at m/z 717 derived from the rosmarinic acid moiety (C_18_H_15_O_7_-), m/z 537 by loss of caffeic acid (m/z 180, C_9_H_8_O_4_), m/z 519 by the loss of a molecule of H_2_O (m/z 18) and McLafferty rearrangement, and m/z 321 by the elimination of the danshensu moiety (C_9_H_10_O_5_).

**Table 2:** Identification of detected phenolics and tanshinones in SMB by UPLC-QQQ-MS/MS

No	Rt	Proposed compound	[M-H]^−^	[M + H]^+^	m/z	Formula	Fragments
**1**	3.405	Danshensu	197	-	198	C_9_H_10_O_5_	197 135 123
**2**	4.909	Protocatechualdehyde	137	-	138	C_7_H_6_O_3_	137 119 108
**3**	9.364	Lithospermic acid	537	-	538	C_27_H_22_O_12_	537 339 293 197
**4**	10.644	Rosmarinic acid	359	-	360	C_18_H_16_O_8_	359 197 179 161
**5**	11.146	Salvianolic acid B	717	-	719	C_36_H_30_O_16_	717 537 519 339 321 175
**6**	12.020	Isosalvianolic acid B	717	-	719	C_36_H_30_O_16_	717 537 519 339 321 175
**7**	28.970	15,16-Dihydrotanshionone I	-	279	278	C_18_H_14_O_3_	279 261 233
**8**	29.531	Trijuganone B	-	281	280	C_18_H_16_O_3_	281 263 235
**9**	29.846	Methuyl tanshinonate	-	339	338	C_20_H_18_O_5_	339 279 261
**10**	30.569	Cryptotanshinone	-	297	296	C_19_H_20_O_3_	297 281 251
**11**	31.350	Tanshinone I	-	277	276	C_18_H_12_O_3_	277 249
**12**	33.759	Tanshinone IIA	-	295	294	C_19_H_18_O_3_	295 267 253

In positive ion mode, six tanshinones were detected and identified. These compounds showed characteristic fragmentation patterns, representing a high abundance of the product ion generated by losing H_2_O from the C ring and cleaving the bond connected to C-4 at the A ring [35]. Based on these characteristic fragmentation patterns, 15,16-dihydrotanshinone I (Rt: 28.970 min, m/z 278, C_18_H_14_O_3_), trijuganone B (Rt: 29.531 min, m/z 280, C_18_H_16_O_3_), methy tanshinonate (Rt: 29.846 min, m/z 338, C_20_H_18_O_5_), cryptotanshinone (Rt: 30.569 min, m/z 296, C_19_H_20_O_3_), tanshinone I (Rt: 31.350 min, m/z 276, C_18_H_12_O_3_), and tanshinone IIA (Rt: 33.759 min, m/z 294, C_19_H_18_O_3_) were detected and identified.

**Fig. 3:**
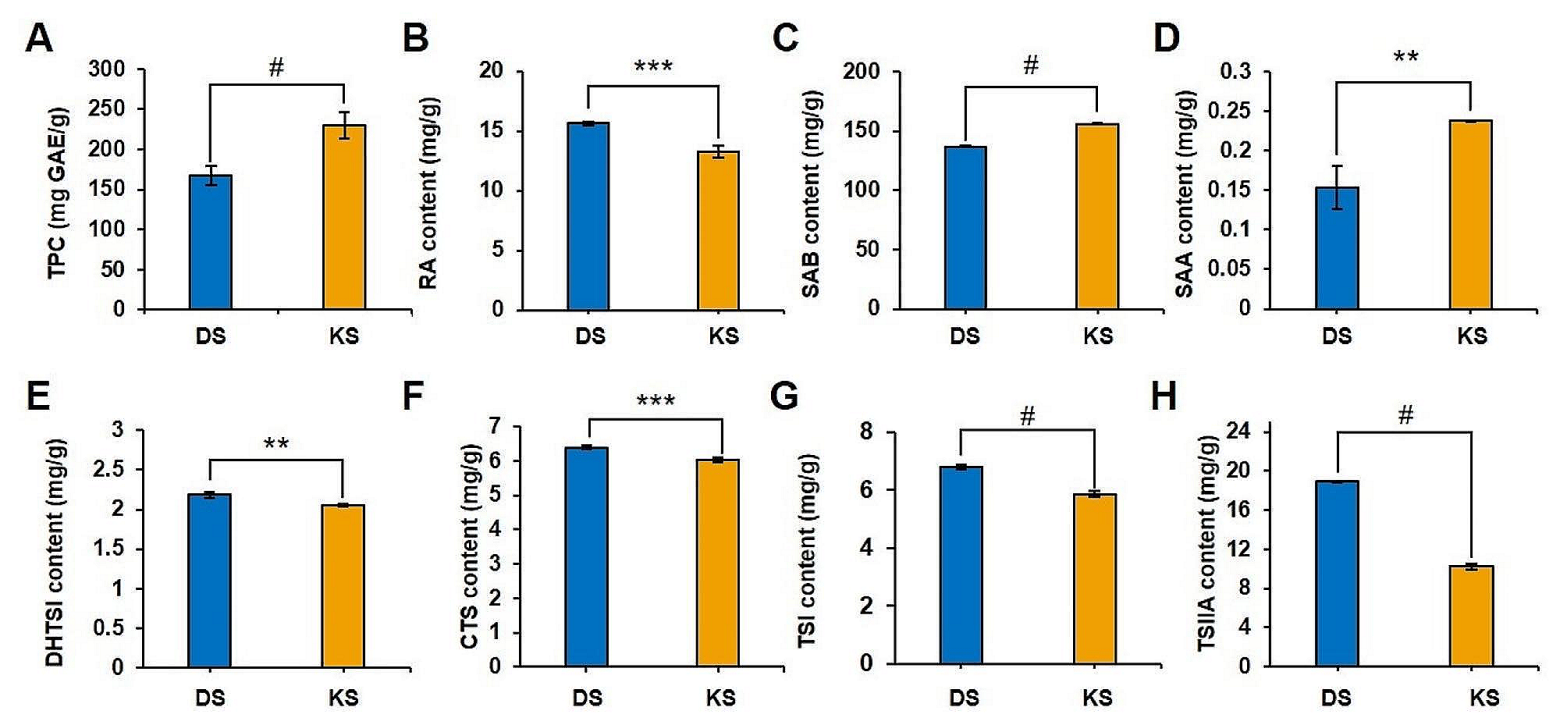
TPC (**A**) was determined by Folin-ciocalteu’s reagent with some modification and calculated by calibration curve generated by gallic acid as the standard. Student’s T-test was conducted to see significant difference between DS and KS and # means *p* < 0.005. Quantification result using HPLC analysis including RA (**B**), SAB (**C**), SAA (**D**), DHTSI (**E**), CTS (**F**), TSI (**G**), and TSIIA (**H**) was done to compare DS and KS. Significant difference was determined by Student’s T-test and expressed following the *p* value as follows: *, *p* < 0.05; **, *p* < 0.01; ***, *p* < 0.005; and #, *p* < 0.001

In the secondary metabolite profiling, 12 compounds were identified. Danshensu, protocatechuic acid, lithospermic acid, and isosalvianolic acid B were identified as minor compounds consistent with previous reports [36]. Consequently, a quantitative analysis of the major components of SMB, including rosmarinic acid (RA), salvianolic acid B (SAB), 15,16-dihydrotanshinone I (DHTSI), cryptotanshinone (CTS), tanshinone I (TSI), and tanshinone IIA (TSIIA) (Fig. 3) were conducted. The quantitative analysis revealed SAB (Fig. 3C) as the primary phenolic compound in both cultivars, with significantly higher content in KS (155.72 ± 0.57 mg/g, *p* < 0.05). This finding aligned with the TPC results (Fig. 3A; DS = 167.46 ± 9.73 mg GAE/g; KS = 229.83 ± 14.45 mg GAE/g). Other reported phenolic compounds, such as RA (Fig. 3B) and SAA (Fig. 3D), were also detected, but their levels were considerably lower in comparison to SAB.

In terms of lipophilic compounds, the primary tanshinone component was identified as TSIIA, with DS containing 18.89 ± 0.11 mg/g and KS containing 10.22 ± 0.31 mg/g. The next highest concentrations were observed for CTS (DS = 6.39 ± 0.05 mg/g; KS = 6.03 ± 0.06 mg/g) and TSI (DS = 6.79 ± 0.08 mg/g; KS = 5.85 ± 0.10 mg/g) in both DS and KS. Consequently, the content of diterpenoid compounds (tanshinones) such as DHTSI (Fig. 3E), CTS (Fig. 3F), TSI (Fig. 3G), and TSIIA (Fig. 3H) were significantly higher in DS (*p* < 0.05).

**Fig. 4:**
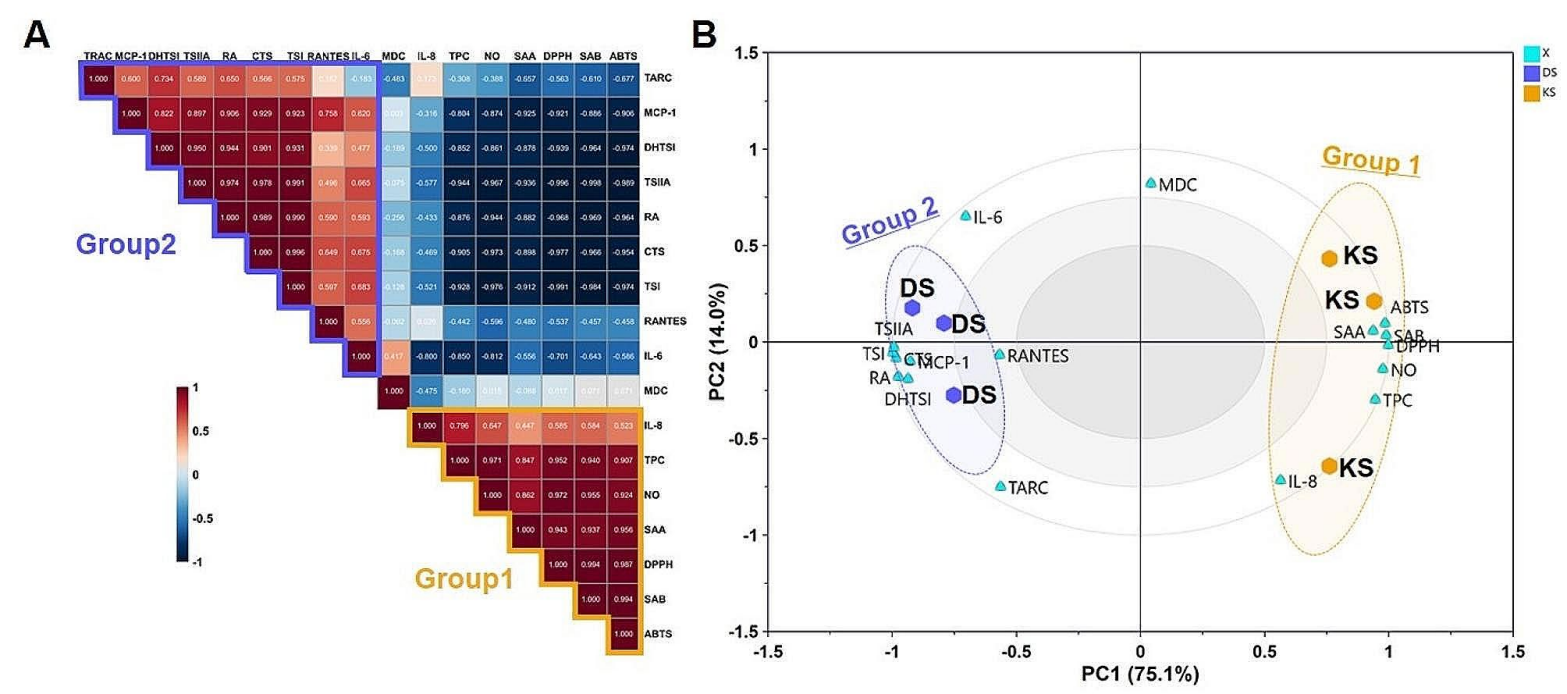
Correlation analysis (**A**) was conducted to evaluate relationships between variables. The Red and blue boxes presented negative and positive correlations, respectively. PCA (**B**) was performed to visualize the relationship among the activities and samples

These results were more clearly explained through statistical analyses, including correlation analysis and principal component analysis (PCA). In the correlation analysis that examined the relationships between factors, two groups (Group 1 and Group 2) were identified. In Group 1, strong positive correlations were observed between components such as TPC, SAA, SAB, antioxidant activity assay results (DPPH and ABTS), and anti-inflammatory activity, showing a correlation Coefficient (*r*) above 0.8. On the other hand, in Group 2, components related to the tannin pathway showed a strong positive correlation with the inhibition of mRNA expression of inflammatory factors observed in the skin cell line for evaluating anti-AD activity (Fig. 4A).

To confirm the associations between these variables and samples, PCA, one of the multivariate statistical data analysis methods, once again validated these results (Fig. 4B). The formed PCA accounted for 89.1% of the total variance in the dataset, with PC1 contributing 75.1% and PC2 contributing 14.0%. Regarding PC1, KS and phenolic components, antioxidant, and anti-inflammatory variables were located in the positive quadrant (quadrants 1 and 3), while DS, tanshinones, and anti-AD variables were located in the negative direction (quadrants 2 and 4).

### Secondary metabolites and pharmacological activities of the fractions

**Fig. 5:**
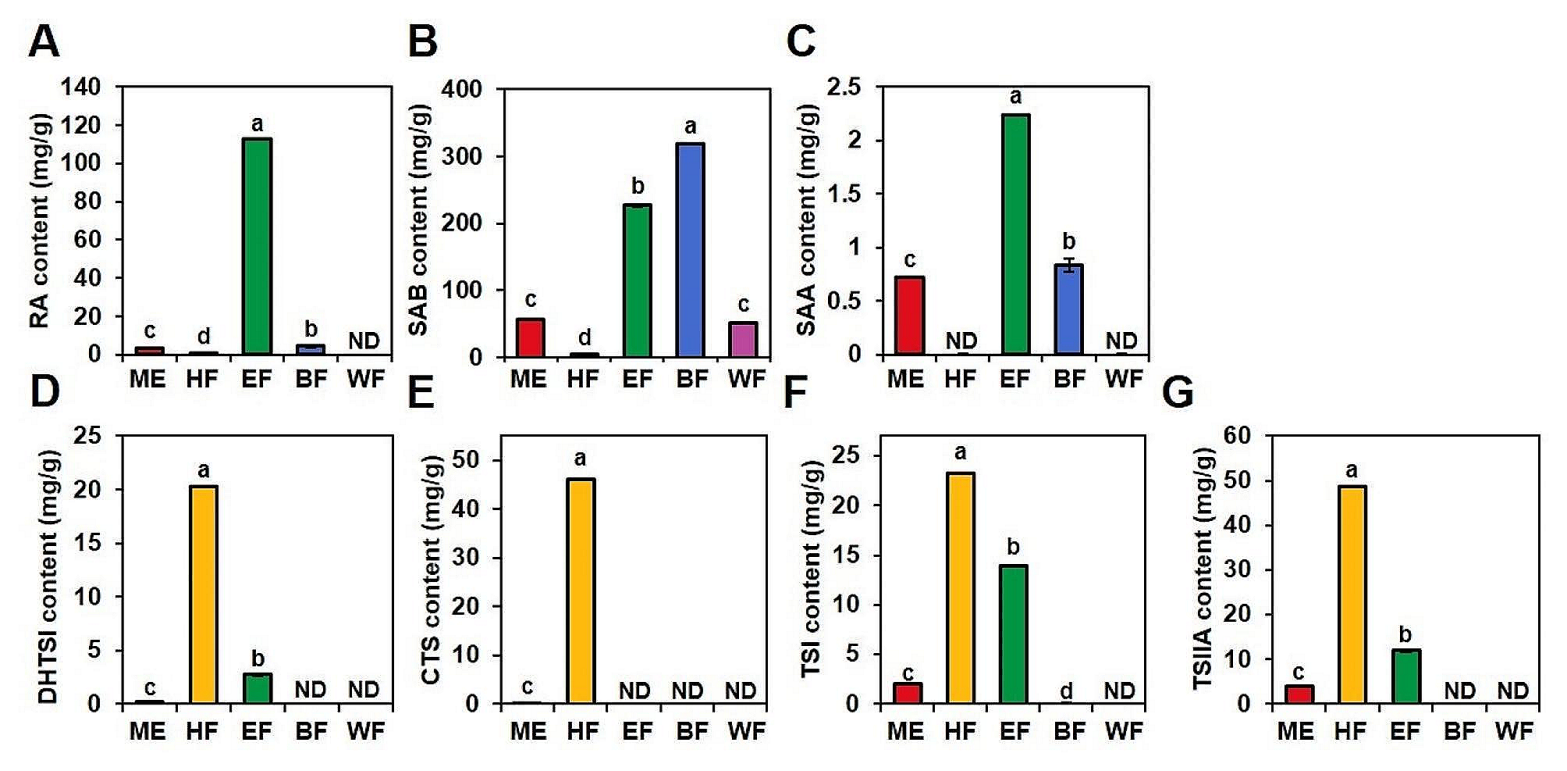
Result of quantification including RA (**A**), SAB (**B**), SAA (**C**), DHTSI (**D**), CTS (**E**), TSI (**F**), and TSIIA (**G**) using HPLC of SMB methanol extract (ME) and fractions including HF, EF, BF, and WF. Quantification data were analyzed by one-pair ANOVA test using Turkey method and different letters (a-d) indicate significant differences at *p* < 0.05. ND, Not detected

Based on the above comparative studies, distinct patterns were observed in antioxidant and anti-AD activities. Fractionation enabled the separation of components with different polarities, as illustrated in Fig. S2. Hydrophilic compounds (RA, SAB, and SAA) were released and partially solubilized in polar solvents (ethyl acetate and butanol), while lipophilic compounds (DHTSI, CTS, TSI, and TSIIA) were solubilized in the non-polar solvent (hexane). These findings were further supported by quantitative analysis shown in Fig. 5. Notably, the EF fraction exhibited a significantly higher content of RA (112.89 ± 0.18 mg/g), while the BF fraction showed the highest content of SAB (317.92 ± 0.54 mg/g), followed by EF (226.69 ± 0.88 mg/g). SAA, a minor phenolic compound, was exclusively detected in EF (2.24 ± 0.06 mg/g) and BF (0.83 ± 0.01 mg/g). In contrast, the HF fraction displayed significantly higher concentrations of tanshinones, including DHTSI (20.32 ± 0.02 mg/g), TSI (23.25 ± 0.05 mg/g), and TSIIA (48.80 ± 0.04 mg/g), and exclusively contained CTS (46.20 ± 0.05 mg/g) among the fractions.

**Fig. 6:**
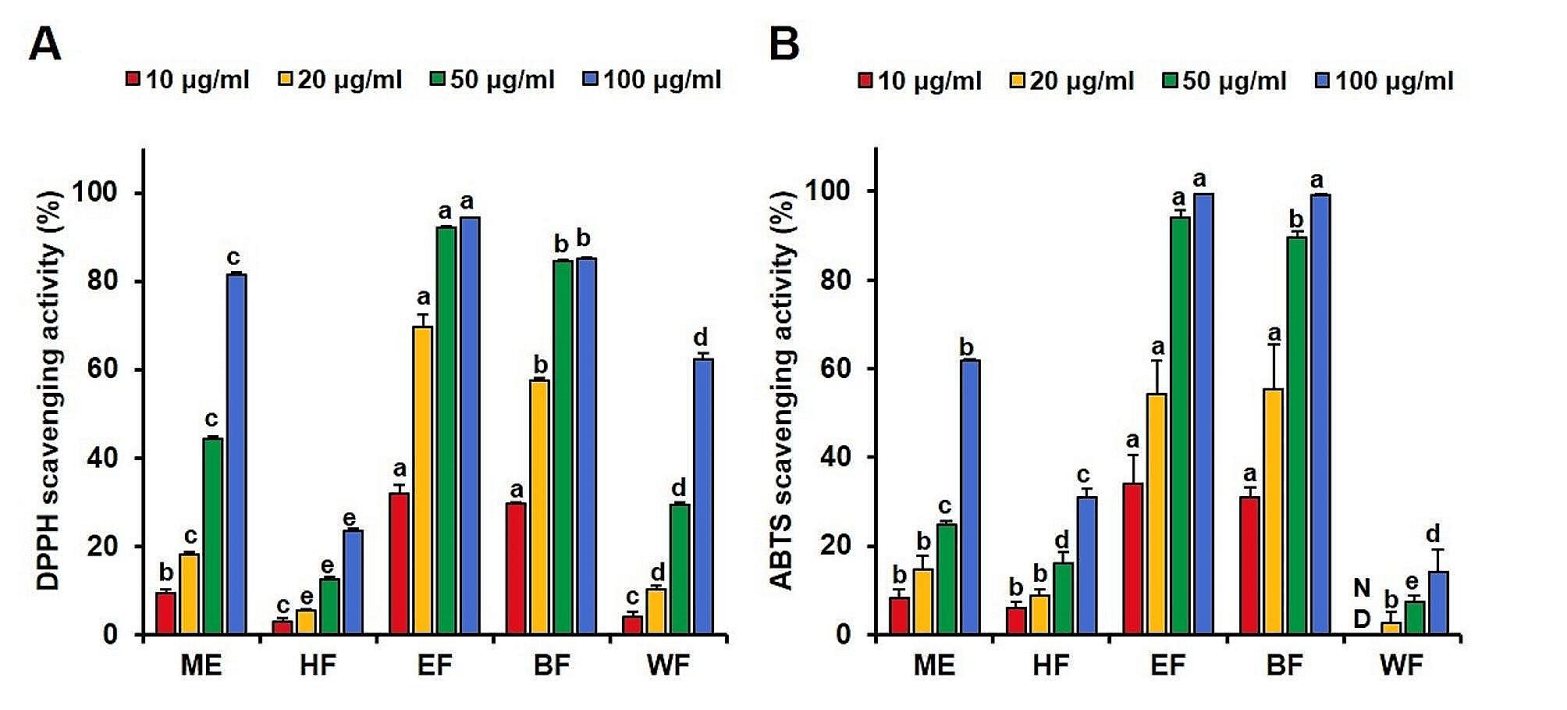
DPPH radical scavenging activity (**A**) and ABTS free radical scavenging activity (**B**) of SMB extract (ME) and fractions (HF, EF, BF, and WF). Data were analyzed by one-pair ANOVA test using Turkey method and different letters (a-e) indicate significant differences at *p* < 0.05. ND: Not detected

**Fig. 7:**
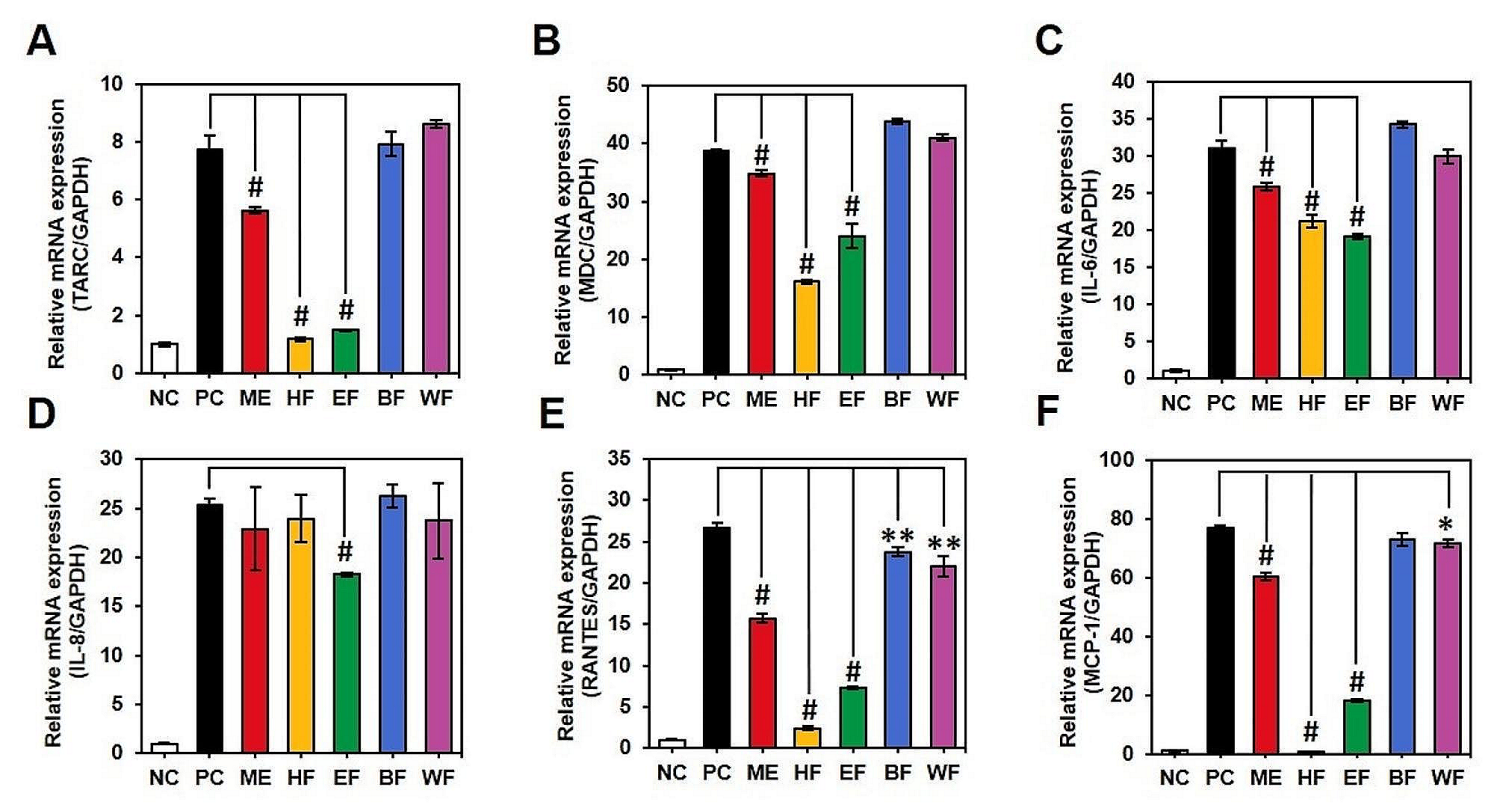
Effect of crude extract of SMB (ME) and fractions (HF, EF, BF, and WF) on TNF-α and IFN-γ-induced chemokines, including TARC (**A**), MDC (**B**), IL-6 (**C**), IL-8 (**D**), RANTES (**E**), and MDC (**F**). Significant difference was determined by Student’s T-test compared to the positive control (the chemokines mRNA expression in the TNF-α and IFN-γ stimulated HaCaT cell) and expressed following the *p* value as follows: *, *p* < 0.05; **, *p* < 0.01; and #, *p* < 0.001

These distinct compound distributions also had a significant impact on the observed activities. Both DPPH radical scavenging activity and ABTS radical scavenging activity were markedly enhanced in the EF (DPPH RC_50_ = 14.7 ± 0.4 µg/ml; ABTS RC_50_ = 9.6 ± 0.7 µg/ml) and BF (DPPH RC_50_ = 17.3 ± 0.2 µg/ml; ABTS RC_50_ = 10.1 ± 0.6 µg/ml) fractions, with lower RC_50_ values indicating higher antioxidant potential (Fig. 6). Regarding the expression of cytokines and chemokines related to AD, the HF layer exhibited the most pronounced effect on influencing the expression of these factors. Remarkably, all extracts significantly suppressed the expression of RANTES (Fig. 7E) (*p* < 0.01). Additionally, the HF and EF samples significantly reduced the expression levels of TARC (Fig. 7A), MDC (Fig. 7B), IL-6 (Fig. 7C), and MCP-1 (Fig. 7F), indicating their potential to suppress AD. Markedly, the HF fraction demonstrated superior anti-AD activity compared to other fractions.

## Discussion

Reactive oxygen species (ROS) involving hydrogen peroxide (H_2_O_2_), hydrogen radical (OH^−^), superoxide anion (O_2_^−^), and nitric oxide (NO) are oxygens containing free radicals [37]. Extremely reactive molecules with unpaired electrons in ROS can oxidative biomolecules and result in cellular damage [38]. In normal conditions, the production of ROS in cells is at low levels and used for the maintenance of cellular homeostasis and function [37, 39] but when the cellular defense mechanism against oxidative stress is impaired by diverse factors, it provokes imbalance between occurrence and extinction of ROS and resulting accumulation of ROS [40, 41]. In abnormal conditions, the enzymatic antioxidants such as catalase, superoxide dismutase (SOD), thiol peroxide, or the non-enzymatic antioxidants including glutathione cannot scavenge over-produced ROS by physiological conditions [42, 43]. This imbalance causes the cytokines expression to increase and consequently, chronic diseases contributed by inflammation occur [44]. Especially, during the inflammation, macrophage (Raw 264.7 cell) activated via the recognition of lipopolysaccharide (LPS) by Toll-like receptors (TLR) produce ROS [45] and stimulate the expression of nitric oxide synthase (NOS) [40]. Among three isoforms of NOS, the inducible NOS (iNOS) has been reported to produce a relatively large amount of NO than other NOS (neuronal NOS, nNOS; and endothelial NOS, eNOS) and provoke the release of inflammatory cytokines [40] such as interleukin-1β (IL-1β), tumor necrosis factor α (TNF- α), interferon-γ (IFN- γ) and IL-17 [40] by activating nuclear factor kappa B (NFκB) pathway [46]. Therefore, NO is a well-known mediator in inflammation and has a relationship with ROS production. In the human body, NF-κB and p38 MAPK mechanisms are the main regulators of inflammatory diseases [47, 48]. In normal conditions, IκB (inhibitor of NF-κB) makes NF-κB exist as the inactivated form in the cytosol [49]. But when the external stimuli induce degradation of IκB (inhibitor of NF-κB), it makes an activated form of NF-κB and induces the expression of diverse pro-inflammatory genes [49, 50]. In addition to the NF-κB mechanism, p38 MAPK is also induced by external stress signals and participates in inflammatory signal transduction and cytokine production. When the TNF-α/IFN-γ that is an important mediator of inflammation is induced in HaCaT cells, p38 and NF-κB act as the contributor against the expression of diverse chemokines including CCL (C-C motif ligand) and CXCL (C-X-C motif ligand), produced by Th cells [51]. It has been proven that receptors of diverse cytokines and chemokines are involved in inflammatory cell infiltration into the lesion and their predominant expression pattern from keratinocytes was differently observed [52]. IL-6 plays a significant role in the acute phase of AD, with increased expression levels produced by macrophages, dendritic cells, and B-cells, and these levels are notably elevated in AD patients [53]. Notably, relatively higher levels of MDC and TARC were also observed in the acute and chronic stages than in the sub-acute stage [54]. MDC and TARC, which are released from lesoined skin, are commonly used as biomarkers of AD due to their positive correlation with severity [55–57]. And as the atopic stage progresses, chemokines are sequentially produced by TNF-α/IFN-γ. RANTES and IL-8, induced by TNF-α, were predominantly observed in the sub-acute stage, while MCP-1 was induced by IFN-γ during the chronic stages [54, 58].

Therefore, the mechanisms underlying antioxidant, anti-inflammatory, and anti-AD activities are interconnected. However, in this study, despite the superior antioxidant and anti-inflammatory activities of KS (Fig. 1), it exhibited lower anti-AD activity (Fig. 2) compared to DS. To interpret these results, a secondary metabolism profile analysis was conducted, and based on this, a quantitative analysis of the major components was also performed. The quantitative analysis (Fig. 3) revealed that DS contained high levels of lipophilic compounds (tanshinones), whereas KS exhibited a high content of SAB, the major phenolics of SMB. Based on statistical analysis, including correlation analysis and PCA (Fig. 4), the differences in compound profiles were expected to contribute to the variations in their respective activities.

To provide evidence for this, fractionation was performed to separate the components based on their polarity (Fig. S3 and Fig. S4). Phenolic compounds corresponding to polar components were predominantly detected in EF and HF (Fig. 5), which was found to be associated with antioxidant activity (Fig. 6). In contrast, lipophilic diterpenoid compounds, such as tanshinones, were quantified at higher levels in HF (Fig. 5), and they exhibited significant activity in factors related to AD (Fig. 7). For the mechanism, ROS and NO have the potential to induce harm to epidermal keratinocytes through mechanisms such as DNA damage, impairment of cellular enzymes, and disruption of cell membrane structures via lipid oxidation [59]. Therefore, it was suggested the preventive effect of SAB on the development of atopy and the inhibitory effect of atopic expression factors of tanshinone components.

## Conclusions

The results obtained in this study suggest a complex interplay among antioxidant, anti-inflammatory, and anti-AD activities. Despite the superior antioxidant and anti-inflammatory activities observed in KS, it exhibited lower anti-AD activity compared to DS. The quantitative analysis revealed that DS had high levels of lipophilic compounds, specifically tanshinones, while KS contained a significant amount of SAB, the major phenolic compounds of SMB. It is hypothesized that these differences in compound profiles contribute to the variations in their respective activities. Fractionation was conducted to reveal these results. The fractionation results further supported these differences, with polar phenolic compounds predominantly detected in EF and HF fractions, correlating with their antioxidant activity, and lipophilic tanshinones quantified at higher levels in the HF fraction, exhibiting significant activity in factors related to AD. Considering the mechanisms involved, reactive oxygen species (ROS) and nitric oxide (NO) can induce harm to epidermal keratinocytes through various pathways, including DNA damage, impairment of cellular enzymes, and disruption of cell membrane structures via lipid oxidation. Therefore, the preventive effect of SAB on the development of atopy and the inhibitory effect of tanshinone components on AD expression factors can be attributed to their ability to mitigate ROS and NO-induced damage.

### Electronic supplementary material

Below is the link to the electronic supplementary material.

Supplementary Material 1

Supplementary Material 2

## Data Availability

The datasets used and/or analysed during the current study are available from the corresponding author on reasonable request.

## Abbreviations

ABTS: 2,2’-Azino-bis-3-ethylbenzothiazoline-6-sulfonic acid
AD: Atopic dermatitis
ANOVA: Analysis of variance
BF: Butanol fraction
CTS: Cryptotanshinone
DS: Dasan
DHTSI: 15,16-Dihydrotanshinone I
DMEM: Dulbecco’s modified Eagle’s medium
DMSO: Dimethyl sulfoxide
DPBS: Dulbecco’s phosphate-buffered saline
DPPH: 2,2-Diphenyl-1-picrylhydrazyl
EF: Ethyl acetate fraction
FBS: Fetal bovine serum
GAE: gallic acid equivalents
HF: Hexane fraction
HPLC: High performance liquid chromatography
INF-γ: Interferon-gamma
IL-6: Interleukin-6
IL-8: Interleukin-8
KS: Kosan
LPS: lipopolysaccharide
MCP-1: Monocyte chemoattractant protein-1
MDC: Macrophage-derived chemokine
ME: Methanol extract
MTT: 3-(4,5-Dimethylthiazol-2-yl)-2,5-diphenyltetrazolium bromide
NO: Nitric oxide
PBS: Phosphate-buffered saline
qRT-PCR: Quantitative real time-polymerase chain reaction
RANTES: Regulated on activation, normal T-cell expressed and secreted
RA: Rosmarinic acid
ROS: Reactive oxygen species
SAB: Salvianolic acid B
SMB: *Salvia miltiorrhiza* Bunge
TARC: Thymus and activation-regulated chemokine
TNF-α: Tumor necrosis factor-gamma
TPC: Total phenolics content
TSI: Tanshinone I
TSIIA: Tanshinone IIA
UPLC-TQ-MS/MS: Ultra performance liquid chromatography-triple quadrupole-mass/mass
WF: Water fraction
