# Supplementary material for: Salvia miltiorrhiza bunge extracts: a promising source for anti-atopic dermatitis activity
Source: BMC Complement Med Ther. 2024 Jun 6;24:217. doi: 10.1186/s12906-024-04524-z (PMC11155122; doi:10.1186/s12906-024-04524-z)
Supplement: Supplementary file 1 — Supplementary Material 1 [file 12906_2024_4524_MOESM1_ESM.docx]

Western blot analysis

HaCaT cells were plated in 6-well culture plates at a density of 1.5 × 10^5^ cells per well and incubated for 48 hours. They were then subjected to serum starvation by using DMEM supplemented with 0.5% FBS and 1% antibiotic solution for 24 hours. Subsequently, the supernatant was replaced with fresh growth media containing different concentrations of samples (0, 2.5, 5 and 10 μg/ml) and incubated for an additional 1 hour. The cells were then stimulated with or without TNF-α/IFN-γ (10 ng/mL each) for 24 hours. After being rinsed thrice with ice-cold DPBS, HaCaT cells were harvested with a scraper and protein extract solution (iNtRON Biotechnology, Seoul, Korea) containing phosphatase inhibitor cocktails and protease. Following centrifugation at 12,000 × g for 15 min at 4°C, the protein concentration in the supernatant was assessed using a DC^TM^ Protein assay kit (Bio-Rad Laboratories, Hercules, CA, USA). Equivalent amounts (20 μg) of protein were separated on 10% SDS-PAGE gels, transferred onto 0.2 μm PVDF membranes (Bio-Rad, Hercules, CA, USA), and blocked with 5% skim milk (dissolved in Tris-buffered saline containing 0.05% Tween 20, TBS-T). The membranes were incubated at 4^o^C with primary antibodies against p-STAT1 (Cell signaling Technology, Beverly, MA, USA, 9167; 1:1000), STAT1 (Cell signaling Technology, 14994; 1:1000), p-STAT3 (Cell signaling Technology, 9145; 1:1000), STAT3 (Cell signaling Technology, 12640; 1:2000), and GAPDH (Cell signaling Technology, 2118; 1:10000). After incubation with horseradish peroxidase-conjugated secondary antibodies (Santa Cruz Biotechnology, Dallas, TX, USA, sc-2357; 1:10000) for 1 h at room temperature. And then rinsing with TBST, the membranes were incubated with enhanced chemiluminescence reagent (Advansta, San Jose, CA, USA). Protein bands were visualized using an iBrightTM CL750 Imaging instrument (Invitrogen, Carlsbad, CA, USA).
