# Supplementary material for: Salvia miltiorrhiza bunge extracts: a promising source for anti-atopic dermatitis activity
Source: BMC Complement Med Ther. 2024 Jun 6;24:217. doi: 10.1186/s12906-024-04524-z (PMC11155122; doi:10.1186/s12906-024-04524-z)
Supplement: Supplementary file 2 — Supplementary Material 2 [file 12906_2024_4524_MOESM2_ESM.docx]

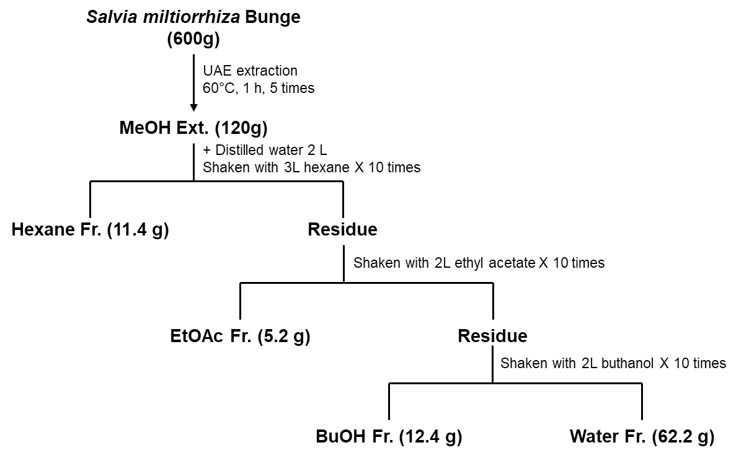
**Supplementary Figures**

**Figure S1**

Sequential solvent fractionation scheme of SMB extract.

**
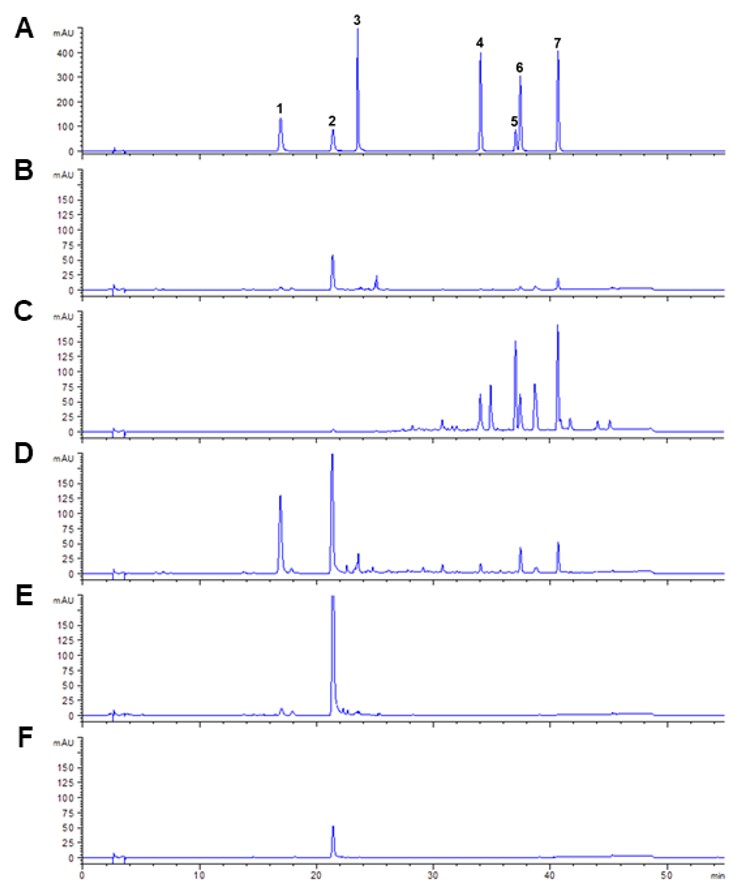
Figure S2**

HPLC chromatogram of standards (A) including RA (1), SAB (2), SAA (3), DHTSI (4), CTS (5), TSI (6), and TSIIA (7) and fractions such as ME (B), HF (C), EF (D), BF (E), and WF (F), respectively.

**
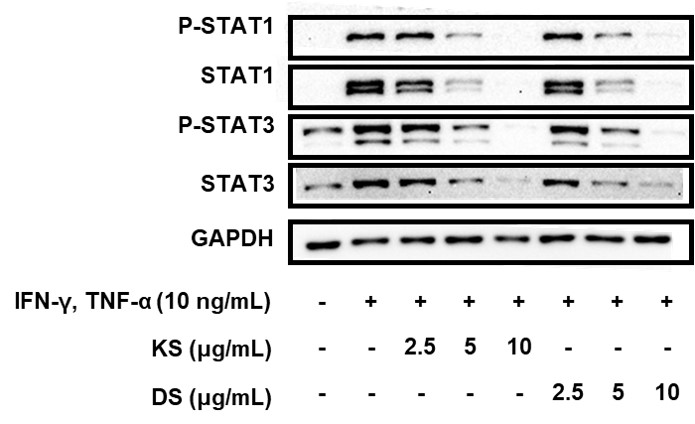
Figure S3**

The effect of DS and KS on IFN-γ/TNF-α-induced activation of STAT1 and STAT3 signaling proteins in HaCaT cells were evaluated by western blot analysis.

**
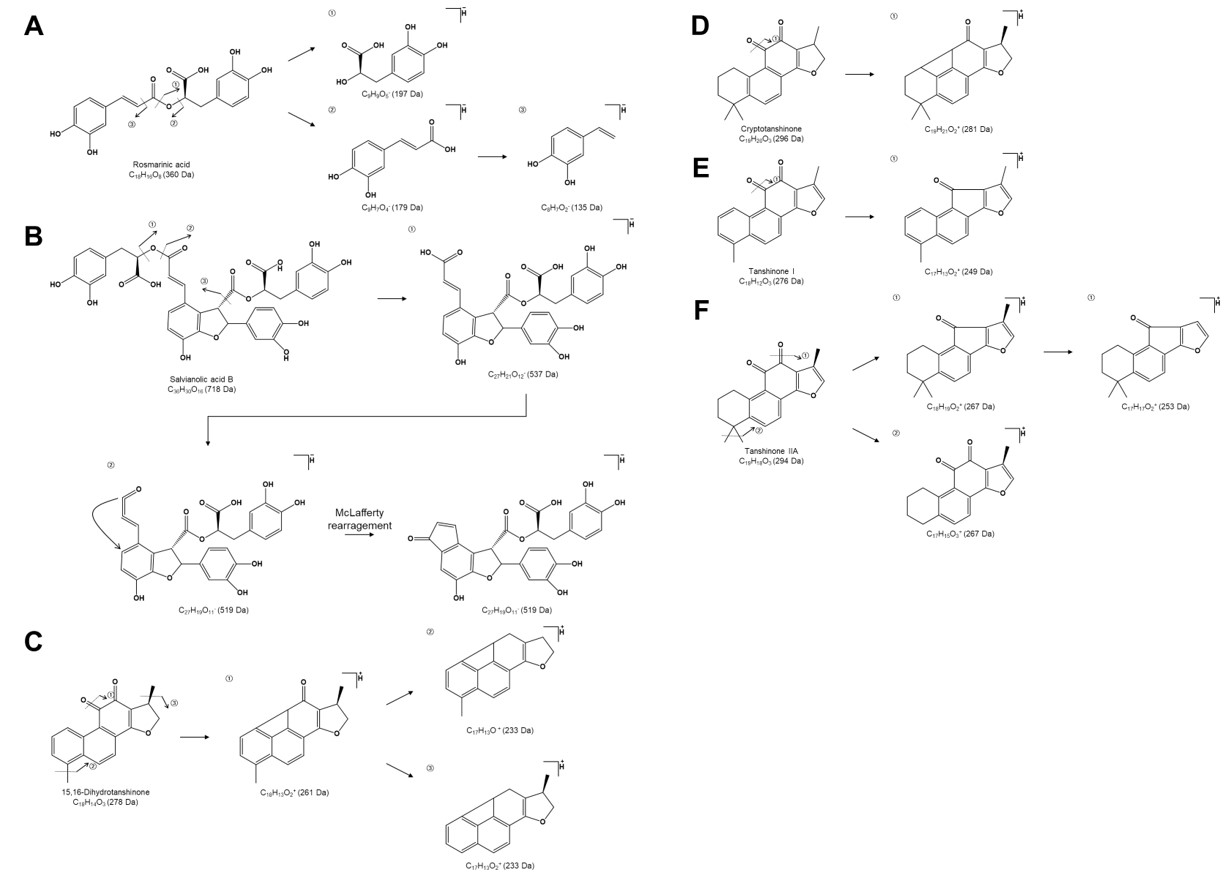
Figure S4**

Fragmentation patterns of detected major compounds in UPLC-TQ-MS/MS including RA (A), SAB (B), DHTSI (C), CTS (D), TSI (E), and TSIIA (F).
